# CREB activity maintains the survival of cingulate cortical pyramidal neurons in the adult mouse brain

**DOI:** 10.1186/1744-8069-2-15

**Published:** 2006-04-26

**Authors:** Hushan Ao, Shanelle W Ko, Min Zhuo

**Affiliations:** 1 Department of Physiology, Faculty of Medicine, University of Toronto Centre for the Study of Pain, 1 King's College Circle, Toronto, Ontario, M5S 1A8, Canada

## Abstract

Cyclic AMP-responsive element binding protein (CREB) activity is known to contribute to important neuronal functions, such as synaptic plasticity, learning and memory. Using a microelectroporation technique to overexpress dominant negative mutant CREB (mCREB) in the adult mouse brain, we found that overexpression of mCREB in the forebrain cortex induced neuronal degeneration. Our findings suggest that constitutively active CREB phosphorylation is important for the survival of mammalian cells in the brain.

## 

Neuronal activity helps to form the complex neuronal circuitry that develops throughout life [1-4]. Among many candidate molecules for mediating activity-dependent plasticity, the transcription factor cyclic AMP-responsive element binding protein (CREB) has been widely investigated. Two major functions of CREB that have been particularly well studied are: (1) its role in the maintenance of long-term memory [2,3] and (2) its role as a survival factor for cells and a molecular transducer for various triggering factors for cell death associated with neurological diseases [5-9]. CREB is rapidly activated by phosphorylation of the serine residue 133 [10]. Inhibition of CREB activity impaired behavioral performance in various memory tests across different species, while the overexpression of CREB facilitated long-term memory [2,3]. The overexpression of dominant-negative CREB or genetic deletion of CREB leads to reduced survival of sympathic/cerebellar neurons and progressive neurodegeneration in postnatal forebrains, while the overexpression of CREB supports neuronal survival [5-9].

The anterior cingulate cortex (ACC) is a key forebrain structure and is implicated in several higher brain functions, including attention, conflict monitoring, memory, pain, pleasure and decision-making [11,12]. Dysfunction of ACC neurons may contribute to the cognitive deficits in mental disorders, including positive and negative symptoms of schizophrenia [13]. CREB activity is increased in ACC neurons by physiological stimulation as well as pathological injury [2,14,15]. To determine if CREB activity contributes to neuronal survival in adult anterior cingulate neurons, we inhibited CREB activity by over-expressing pCMV-CREB133, a dominant negative CREB (mCREB), in the ACC of adult mice using a newly developed method of microelectroporation [16].

To determine if mCREB was actually expressed in cortical neurons, we first performed microelectroporation of the GFP tagged mCREB (GFP-mCREB) into the mouse ACC. Consistent with a previous study, which overexpressed a mutant form of calmodulin (CaM) in the ACC [16], cells transfected with GFP-mCREB were found in the area surrounding the injection site (n = 8 mice; Fig. 1A). Next, we wanted to determine if inhibiting CREB activity, by overexpressing mCREB, might affect the survival of cingulate neurons. In order to quantify the loss of cortical pyramidal cells, we used YFP transgenic mice that selectively express YFP in cortical pyramidal neurons [17]. Interestingly, the overexpression of mCREB led to a significant loss of pyramidal neurons in the ACC (n = 10 mice; Fig. 1B). YFP fluorescence was reduced at the transfected area (n = 10 mice; mean, 66%; ranging from 30% to 92% reduction). Since microelectroporation was performed on only one side of the ACC, a direct comparison of the effects of CREB could be made within the same animal. Experiments were also performed using the pCMV vector alone to determine if it had any effect in the absence of the expression of mCREB. No neurodegeneration was found in these animals (n = 5 mice). Additional staining experiments were performed to examine cytopathological features of apoptosis in degenerating cortical neurons that were transfected by mCREB. Hoechst nucleus staining showed that the nuclei had chromatin condensation, pyknosis, and fragmentation (n = 5 mice, Fig. 1C). Caspase 3 staining showed abundant caspase-3 positive cells at the mCREB transfection area, as compared to the wild-type mouse (n = 10 mice, Fig. 1C).

**Figure 1:**
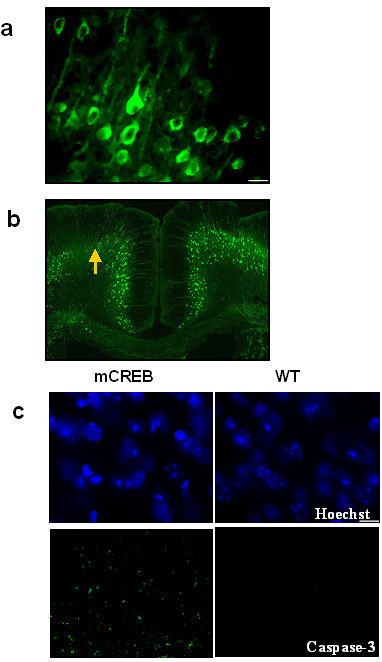
**Effects of overexpressing mutant CREB in adult cingulate neurons**. a. Transfected ACC area showing GFP tagged mCREB protein expression. Scaled Bar: 10 μm. b. YFP mouse brain transfected with the CREB vector. The pCMV transfected brain showed no neurodegeneration (right). The mCREB transfected area showed significant neurodegeneration (left). c. Hoechst nucleus staining showed nuclear condensation and fragmentation at the mCREB transfected area (Top). Caspase 3 staining showed that apoptosis occurred at the mCREB transfected area (Lower).

Our results provide direct evidence that CREB activity plays a critical role in the survival of adult cingulate cortical neurons. The expression of mCREB induced significant apoptosis at the mCREB transfection area. The ability of mCREB to initiate programmed cell death in cortical neurons is consistent with CREB's role in survival and apoptosis described in *in vitro *studies [5-9]. Loss of neurons due to inhibition of CREB activity may provide a molecular mechanism for cellular loss in forebrain regions related to various mental illnesses. Furthermore, drugs targeted at the CREB signaling pathways may help to prevent or rescue neuronal loss in the brain.

## Materials and methods

Surgical procedures were performed in sterile conditions and were approved by the Animal Care and Use committees of Washington University School of Medicine and the University of Toronto. For microelectroporation, a Grass SD9 stimulator was used to deliver square wave electric pulses. A pair of silver electrodes were placed 3 mm anterior and 2 mm posterior to the injection site, respectively (2.5 mm depth). The animals were placed in a Kopf stereotaxic apparatus fitted with a mouse adaptor. Microinjections of DNA into the ACC were performed at the following coordinates: 0.7 mm anterior to the Bregma, 0.4 mm to the midline, and 1.8 mm depth from the surface of the skull. The pCMV-CREB133 (0.8 μl) and pCMV vector DNA (0.5 μg/μL) were injected at each site at a rate of 0.05 μl/min, using a 30 gauge needle with cannula tubing connected to a Hamilton syringe. Adult YFP (yellow fluorescent protein) mice (n = 15 mice; 23–26 g, generously provided by Dr. Sanes [17]) were used. Ten YFP mice were microinjected with pCMV-CREB133, and 5 YFP mice were microinjected with pCMV vector. Wild-type (wt) pCMV-CREB vector and mutant CMV-CREB133 vector were purchased from Clontech (Cat 6014-1, BD Biosciences Clontech, Palo Alto, CA). The pCMV-CREB vector constitutively expresses the human wild-type (wt) CREB protein and the pCMV-CREB133 vector expresses a mutant variant of the human CREB protein that contains a serine to alanine mutation corresponding to amino acid 133. This mutation blocks the phosphorylation of CREB. The fusion of the GFP to the N-terminus of mCREB allowed us to determine the level of protein expression.
